# Radiation Therapy as a Modality to Create Abscopal Effects: Current and Future Practices

**DOI:** 10.7759/cureus.7054

**Published:** 2020-02-20

**Authors:** Dennis Adjepong, Bilal Haider Malik

**Affiliations:** 1 Neurological Surgery, California Institute of Behavioral Neurosciences and Psychology, Fairfield, USA; 2 Internal Medicine, California Institute of Behavioral Neurosciences and Psychology, Fairfield, USA

**Keywords:** radiation therapy, abscopal response, tumor cells, cytotoxic, irradiated tumor

## Abstract

In our empathetic understanding of abscopal effect (AbE), research has shown that the immune system is stimulated by radiation, which results in the formation of an AbE. The AbE is referred to as a response from the irradiated volume. Despite the existence of key gaps in our understanding, there is an urgent need to explore what the underlying effect is. The aim of this article is to argue neurosurgeons and the healthcare practitioner's knowledge of the AbE. Our goal is to identify more gaps in our understanding of the AbE and seal other gaps as well. This study will review medical journals and bring together the most updated information related to AbEs.

## Introduction and background

Radiation therapy (RT) is a significant procedure in the treatment and management of malignant tumors; hence, it is used to treat about 50% of the cancer patients [1]. Its primary mechanism is damaging the solid tumor cells through autophagy, senescence, and apoptosis, which are approached in clinical ionizing radiation [2]. In the past, RT was an immunosuppressive process due to its effects on the leukocytes caused by the cytotoxic effects; for example, the lymphopenia phenomenon was common among patients who suffered from solid tumors, including lung cancer, neck tumors, and breast cancer [3]. In recent years, RT has been recognized as a procedure to induce and activate the immune system through tumor regression in distant non-radiated tumor sites, making it significant in anti-tumor and immune-mediated responses [4]. The impact of RT on distant irradiated tumor sites results in a phenomenon known as the abscopal effect (AbE) [5]. The impact of AbE on the immune system was first identified in 2004, in an experiment with T cell-deficient mice, where the results showed the absence of the AbE [6]. Although studies documenting the clinical relevance of the AbE are rare, recent studies on immunogenic effects and biological mechanisms of both RT and the AbE have introduced a different perspective on the possible clinical benefits of RT [7]. The damaging of associated molecular patterns caused by immunogenic cell death (ICD) after local RT results in an improved presentation of antigen in the cytotoxic immune system [8].

## Review

Methods

The main goal of the presented study is to access concepts of RT concerning immunotherapy, their molecular effects, and AbEs in tumor microenvironments. Relevant clinical research from current peer-reviewed sources was utilized. Relevant literature exploring RT as a modality to create AbEs was used, with the study taking advantage of scientific analysis and clinical trials to make conclusions and identify the existing literature gap.

Results

AbEs evident after RT were reported in about 94 cases of a total of 52 articles used in the literature review. With or without the immunotherapy, 48 incidents were repeated trials, with the report of the timeline of the article between the years 1970 and 2019. Forty-seven of the cases were treated using RT alone, whereas the rest used radiation in addition to immunotherapy. Twenty-four cases of abscopal responses occurred among patients with RT immediately after undergoing RT.

Abscopal effects - current and future practices

A combination of immunotherapy and radiation is considered an exciting avenue of clinical and pre-clinical investigation [9]. Synergies between the two expand the role of RT in local therapy. AbE is considered to be the tumor regression located outside the irradiated field [10]. It is regarded as a phenomenon that is enhanced by checkpoints and mediated by lymphocytes blockades. There are limited cases of reports with radiographic regression and extracranial abscopal responses after treatment with brain metastases with therapy. Observations show that AbEs traverse into the blood-brain, hence becoming a barrier [11]. This case demonstrates the role of radiations in ensuring the release of disruptions to the blood-brain barrier and tumor-associated antigens [12]. AbEs are mainly sporadic events of tumors that begin to grow following RT treatments as observed through irradiated sites from a distance [13]. Mechanisms often have several origins, including infection by inflammatory agents such as cytokines, the distance effects, and the secondary immunity to devices [14].

AbE is described to be the regression of the tumor, lesions, or metastatic regions located outside the radiation field [15]. These AbEs, over time, observed as immunogenic tumor entities. Systematic reactions to anti-tumors after RT have mostly resulted in the regression of tumors, with AbEs [16]. AbEs are considered effective, given the anti-PD-1 irradiation and treatment. AbEs explore regressions in tumors and lesions, including metastatic regions induced by radiation. It is also essential to enhance increased radiation dose, site timing, and irradiation, and fractionations with other systematic therapies resulting in reduced metastases and tumors [17]. Radioimmunotherapy (RIT) hence guarantees accurate measurement, schedule, and checking of radiological images effectively [18]. AbE induction is also considered useful, given its effectiveness in immunogenic and optimal immunogenic responses. 

Biochemistry of disease

Biological research is focusing on the underlying impact of the AbE major on tissue re-oxygenation, cellular repopulation, repair pathways, and DNA damage. RT results in the damaging of DNA on a molecular level through the induction of radicals [19]. Destruction of cells through RT resulted in release or exposure of damage-associated molecular pattern molecules (DAMP) such as adenosine triphosphate (ATP), High Mobility Group Protein B1 (HMGB1), and plasma membrane-exposed calreticulin [20]. In standard treatment of metastatic disease, chemotherapy administration targets biologic agents and hormonal therapy [21]. RT also triggers the death of bystander cells through the secretion of proinflammatory cytokines or reactive nitrogen or oxygen species [22].

Economic cost to healthcare industries

The process of using RT as a modality in creating AbEs has always resulted in decreased costs for healthcare industries, patients, and insurance companies [23]. This has also resulted in a reduced stay at hospitals, with surgeons and healthcare practitioners utilizing technology advancement to achieve higher level and quality images during the diagnosis and treatment processes. Doctors and patients are also able to manage their patient's health and well-being better, guaranteeing the success of the therapy process [24]. Below is a table ( table 1) showing healthcare cost per year on using RT and AbEs.

**Table 1:** Economic savings to healthcare industries per year on using radiation therapy and abscopal effects

Year	Author	Healthcare Cost Saving	Inferences
2014	Levy et al. [21]	$4500 per month	$54000 annually
2015	Gui et al. [8]	$3675 per month	$44100 annually
2016	Brenneman et al. [22]	$2392 per month	$28704 annually
2017	Aboudaram et al. [23]	$7630 per month	$91560 annually
2018	Rodríguez et al. [13]	$2900 per month	$36800 annually
2019	Yilmaz et al. [17]	$7644 per month	$92728 annually

Clinical trials with abscopal effects of tumors

Below are numerous tumors that exhibited AbEs. The table below summarizes the kind of RT used and its associated tumor and whether the AbE occurred ( Table 2).

**Table 2:** Abscopal effects exhibited by numerous tumors and the kind radiotherapy dosage administered

Studies	Author name	Tumors	Year	Radiotherapy	Occurrence of abscopal
10	Kwon et al. [24]	Metastatic prostate cancer	2014	Bone-directed radiotherapy	Not-Identified
41	Golden et al. [25]	Metastatic solid tumors	2015	Fractions- 35 Gy/10	Identified
10	Levy et al. [21]	Metastatic tumors	2016	Fractions- 28 Gy/5	Not-Identified
17	Aboudaram et al. [23]	Melanoma	2017	Fractions- 30 Gy/10	Identified
70	Koller et al. [16]	Melanoma	2017	Stereotactic radiosurgery	Identified
10	Hamilton et al. [2]	Metastatic lung cancer	2018	Stereotactic radiosurgery	Identified
15	Rodriquez et al. [13]	Colon cancer	2018	Stereotactic ablative	Identified
23	Vatner et al. [9]	Metastatic breast cancer	2018	Fractions - 22.5 Gy/3	Identified
9	Brenneman et al. [22]	Metastatic retroperitoneal sarcoma	2019	Proton beam radiotherapy	Identified
11	Barsky et al. [7]	Malignant pleural mesothelioma	2019	Fractions- 30 Gy/10	Identified

Unanswered questions

In this study, various important questions need to be addressed, including how to monitor and determine the abscopal responses. There is also a gap in how other modalities, including particle radiation such as carbon ions or protons, electroporation, and radio ablation, impact the AbE. The study identified the need to research the best disease site, which should be irradiated to acquire a maximal abscopal response.

## Conclusions

The study explores diverse criteria depicting regression in lesions located off the irradiation field. The identification of AbE after radiation also has distinct treatment options and effects of immunotherapy. Notably, healthcare practitioners and surgeons are concerned with the patient's health, believing that RT as a modality in creating AbEs helps improve patient health and outcomes. The process guarantees a more precise identification of AbE inductions. Further studies are also expected to improve the sequencing of RIT with RT as a modality in creating AbEs to enact appropriate approaches during the diagnosis and treatment processes to achieve optimal immunogenic responses.
